# Editorial

**Published:** 1838-09-12

**Authors:** 


### THE MEDICAL EXAMINER.

PHILADELPHIA, SEPT. 12, 1838.

The following notice of the yellow fever, now prevailing at Charleston, S. C., is extracted from a letter written by Dr. De Saussure, of that city, to a medical friend now at Philadelphia. We hope to receive a more detailed account of the symptoms, treatment, and statistics of this fever from the same source; in the meanwhile, our readers will find this notice of the fever extremely interesting—coming as it does from a well educated and careful observer.

"Charleston, August 31, 1838.

"Native children are now beginning to be attacked. The disease has not, however, increased as rapidly as its commencement threatened. The first cases were fatal to a most alarming degree. Out of twenty or thirty which I either saw or heard of, not one recovered. Of these, one was a patient of my own, a young man aged twenty-three. Since that time, the disease has varied very much; some of the cases are distinct yellow fevers of a single paroxysm, others assume a remittent and milder form. I find great difficulty in taking notes of the cases, as the patients enter the hospital after the fever has gone off, and then there is scarcely any thing to note; gradual prostration, injected eyes, yellow skin, and black vomit forming all the symptoms.

"I have seen no petechiæ, rose coloured spots, or sudamina. In most of the first cases a single paroxysm of fever was distinctly marked with violent pain in the head, back, and limbs, injected eyes, great oppression at the præcordia, and, sometimes, irritability of stomach. Of these symptoms, the three first are especially characteristic of the disease. After the hot stage, which lasts from ten to seventy, or eighty hours, the fever goes off, leaving a hot skin, slow, small pulse, great thirst, and injection of the eyes, anxiety of countenance, tongue dry, red, and swollen, covered with a dark fur, and, sometimes, delirium. Black vomit now comes on, with numerous stools, consisting of a similar liquid. The pulse sinks, the skin becomes cold and of a dark yellow colour; delirium goes off, the patient feels easier, lies quietly on his back, says he is well, and dies. On examination after death, we find violent inflammation of the stomach, sometimes engorgement of the liver; the gall bladder filled with a greenish black bile, of the consistence of tar; the other organs are healthy, but the blood is very fluid and has entirely lost its consistence.

"I have not seen a case of typhus fever. Those which are sometimes called here by that name, are malignant remittent fevers, without delirium, injected eyes, or foul tongue. There is no diarrhœa, and no change in the appearance of the skin. In several of the cases, the fever subsided and left the patient apparently convalescent. At the end of two or three days it returned, accompanied by diarrhœa and sudamina, and, in one case, the diarrhœa was attended with such copious discharges of blood from the bowels as quickly to destroy life."

